# Yuck, This Biscuit Looks Lumpy! Neophobic Levels and Cultural Differences Drive Children’s Check-All-That-Apply (CATA) Descriptions and Preferences for High-Fibre Biscuits

**DOI:** 10.3390/foods10010021

**Published:** 2020-12-23

**Authors:** Pernilla Sandvik, Monica Laureati, Hannah Jilani, Lisa Methven, Mari Sandell, Marlies Hörmann-Wallner, Noelia da Quinta, Gertrude G. Zeinstra, Valérie L. Almli

**Affiliations:** 1 Department of Food Studies, Nutrition and Dietetics, Uppsala University, 75236 Uppsala, Sweden; 2 Department of Food, Environmental and Nutritional Sciences, University of Milan, 20133 Milan, Italy; 3 Institute for Public Health and Nursing Research—IPP, University of Bremen and Institute for Preventions Research and Epidemiology—BIPS, 28359 Bremen, Germany; jilani@uni-bremen.de; 4 Department of Food and Nutritional Sciences, University of Reading, Whiteknights, Reading RG6 6AH, UK; l.methven@reading.ac.uk; 5 Department of Food and Nutrition, University of Helsinki, FI-00014 Helsinki, Finland; mari.sandell@helsinki.fi or; 6 Functional Foods Forum, University of Turku, FI-20014 Turku, Finland; 7 Institute of Dietetics and Nutrition, University of Applied Sciences FH JOANNEUM, 8020 Graz, Austria; marlies.hoermann-wallner@fh-joanneum.at; 8 AZTI, Food Research, Basque Research and Technological Alliance (BRTA), 48160 Derio, Spain; ndaquinta@azti.es; 9 Food, Health & Consumer Research, Wageningen Food & Biobased Research, 6708 Wageningen, The Netherlands; gertrude.zeinstra@wur.nl; 10 Department of Innovation, Consumer and Sensory Sciences, Nofima, N-1430 Ås, Norway; valerie.lengard.almli@nofima.no

**Keywords:** food neophobia, cross-cultural, penalty analysis, preference mapping, preadolescents

## Abstract

Food neophobia influences food choice in school-aged children. However, little is known about how children with different degrees of food neophobia perceive food and to what extent different sensory attributes drive their liking. This paper explores liking and sensory perception of fibre-rich biscuits in school-aged children (*n* = 509, age 9–12 years) with different degrees of food neophobia and from five different European countries (Finland, Italy, Spain, Sweden and United Kingdom). Children tasted and rated their liking of eight commercial biscuits and performed a Check-All-That-Apply task to describe the samples and further completed a Food Neophobia Scale. Children with a higher degree of neophobia displayed a lower liking for all tasted biscuits (*p* < 0.001). Cross-cultural differences in liking also appeared (*p* < 0.001). A negative correlation was found between degree of neophobia and the number of CATA-terms used to describe the samples (*r* = −0.116, *p* = 0.009). Penalty analysis showed that degree of food neophobia also affected drivers of biscuit liking, where particularly appearance terms were drivers of disliking for neophobic children. Cross-cultural differences in drivers of liking and disliking were particularly salient for texture attributes. Further research should explore if optimizing appearance attributes could be a way to increase liking of fibre-rich foods in neophobic children.

## 1. Introduction

Food neophobia is considered one of the strongest predictors of the number of foods liked and tried in school-age children [1,2]. It has also been associated with decreased dietary variety and a less varied range of food preferences, especially with regard to healthy foods [3,4,5]. In particular, fibre intake has been shown to be lower among food neophobic children [6]. Food neophobia, defined as the rejection of new and unfamiliar food, usually starts in the second year of life [7]. This behavioural trait is considered as a developmentally appropriate response against the ingestion of new and potentially toxic foods [8]. The trajectory of the behaviour is not clear but food neophobia has been described to peak between 2–6 years of age [7], however for some subjects it is a more persistent trait [9]. Although the absolute individual degree of neophobia most often decreases with age, the relative degree of neophobia compared to other children may be stable [10]. At any given age, the degree of neophobia widely varies between children, with some of them even displaying a food neophilic behaviour [11,12,13].

In addition to the important role of age, other biological and environmental factors have been associated with food neophobia [14]. Using parent reported questionnaires, several studies have shown child food neophobia to be associated with higher levels of visual, tactile, smell and taste sensitivity [3,15,16]. A recent study on adults with different degrees of neophobia however, showed no difference in chemosensory responses between the groups [17]. In 10-year olds, tactile sensitivity and taste sensitivity have been associated with selective eating [15]. Results on the role of general visual sensitivity (measured with statements such as e.g., “Covers eyes, or squints to protect eyes from light”) in food neophobia have been mixed [15]. Still, visual cues and probably the cognitive expectations of what these cues mean are important drivers of neophobic reactions. To increase the chance of acceptance, the food should be visually familiar down to the details [16]. Food neophobia and pickiness have been reported to depend partly on preference for certain perceptual properties of food such as colour, visually perceived texture, shape, smell [18]. On the contrary, less exploration of the chemosensory environment due to a more restrictive sniffing behaviour has been described as associated with food neophobia [19]. It is unclear if the higher degree of anxiety that has been associated with neophobia [15], coupled with the potential increased sensory sensitivity, may induce a less or a more analytical approach towards the foods′ sensory characteristics due to the risk of negative sensory experiences. Farrow and Coulthard [20] put forward Kahnemans’ [21] theory on decision making and hypothesized that food neophobic children rely more on a fast, intuitive and automatic decision-making route (system 1) when deciding to taste or not to taste a food as compared to a slower, effortful and informed route (system 2). To date, it is unknown whether variations in degree of neophobia are reflected in children’s cognitive sensory perception and description of foods. Further, there is a scarcity in research with regard to how children with different degrees of food neophobia sensorially perceive food and what role different sensory attributes play in liking.

Cross-cultural differences are known to often impact consumer perception and preferences with regard to foods in adults [22], however not systematically [23]. Estay et al. [24] recently reported larger effects of culture than effects of gender and age on liking for vegetables in Chinese, Northern American and Chilean children, while Zhang [25] reported no cultural differences in Northern American and Chinese children’s preferences for package designs. Few cross-cultural studies on school children’s characterisation and preference for food samples have been conducted within Europe (for a study on taste, see Lanfer et al. [26]). Therefore, little is known on the possible uniformity or differences of their perception.

Children are increasingly involved in sensory and consumer testing with the aim of developing food products targeted to them and/or better understanding the processes influencing food preference and choice [27]. The Check-All-That-Apply (CATA) method is commonly used to define which sensory attributes consumers perceive in food products [28]. Although less frequently used with children, the CATA method has shown to be an appropriate, child-friendly approach to get insights on how children perceive food products as well as to identify the most relevant sensory attributes that affect children’s hedonic perception [24,29,30,31]. The focus of the present study is to explore the role of individual characteristics such as food neophobia and cultural background for describing foods. Knowledge of how food neophobia status and country-related differences affect children’s CATA descriptions and preferences is relevant from a methodological perspective in design and interpretation of sensory data with children, but also from a practitioners’ perspective. In fact, a better understanding of which sensory attributes are most commonly perceived and how they affect liking is relevant to product development and interventions for encouraging consumption of healthy food products among children with food neophobia.

The aim of this paper is to explore differences in liking and sensory perception of fibre-rich biscuits in a cross-cultural sample of children with different degrees of food neophobia. The Check-All-That-Apply (CATA) method is used for descriptive data collection. We hypothesize that degree of food neophobia and country of residence will affect 9–12-year olds’ liking of biscuits and their use of CATA-terms when describing the biscuit samples.

## 2. Materials and Methods

### 2.1. Participants

This paper is based on a cross-sectional study including 509 children aged 9–12 years, recruited via primary schools in five European countries (Finland, Italy, Spain, Sweden and United Kingdom). Children from Austria were also included in the original data collection but were excluded in the present study due to low validity and reliability on the measurement of child food neophobia. In the chosen age span, reading skills are sufficient for most self-administered tasks and complex evaluation tasks can be performed [27]. The study protocol was approved by the relevant research ethics committee of each country, and written consent was obtained from the parents according to the declaration of Helsinki (Austria: No. 30-200 ex. 17/18, Finland: No. 12/2018, Italy: No. 49/17, Spain: No. PI2017180, Sweden: 114 No 2017/549, UK: No. UREC 18/15). Children were informed orally and gave their oral consent to participate. Children lacking parental consent and children with allergies either did other activities during the test or received allergy-friendly dummy samples (data excluded from the study). Here, concern was taken to involve everyone into activities.

### 2.2. Biscuit Samples

Biscuits were chosen to be used as test products because they are well accepted by children, the sensory quality of biscuits is stable over time and easy to distribute to the different countries involved in the study. Eight commercial biscuits from an Italian company were used (Table 1). Most of the biscuits belonged to a fibre-enriched product range containing between 2.8–10.0 g of fibre per 100 g. A pilot test was performed with 4–10-year-old children in five countries to test the suitability of the samples. None of the biscuits was particularly rejected or liked by the children and all 8 biscuits were possible to test in one single session without causing any fatigue.

### 2.3. Generation of Check-All-That-Apply (CATA) Terms

CATA terms were generated in a word elicitation task with 66 children of the target age group (8–11 years, 45% girls). The aim was to ensure a broad range of descriptive words for the sample set as well as a good understanding of the CATA terms included in the main test by the subjects. In order to capture cultural differences, the word elicitation task was conducted in four countries representing Northern (Sweden, *n* = 14), Central (Austria, *n* = 11 and UK, *n* = 17) and Southern (Italy, *n* = 24) Europe. The elicitation task was performed in an interview setting with one child at a time. Each child received three biscuits according to an incomplete balanced block design so that all samples were covered across children. The child was asked to look, smell, feel and taste the biscuits successively and to describe them. When the first spontaneous descriptions were over, the children were asked to describe similarities and differences between the samples in order to facilitate elicitation of extra words, in a repertory grid-like approach [32]. All elicited terms including descriptive, hedonic and usage words were recorded (*n* = 473), however only sensory descriptive words were retained for the present study (*n* = 354). Most of the elicited terms belonged to appearance (34.6%), thereafter taste (25.4%), smell (20.7%) and texture (19.2%). In Sweden, the participating children elicited on average 10.0 sensory terms, in UK 7.4, in Austria 6.9 and in Italy 5.5 terms per child. The descriptive words were translated to English and categorized according to sensory modality by the experimenters. Terms were selected for the CATA test of the main study as follows: (i) sensory terms cited by at least 8% of all children were included, except for non-discriminative terms such as Round (18.0% citations) and Brown (9.1%) which applied to all biscuits, (ii) texture terms Dry (7%), Grainy (5.6%), Smooth in mouth (5%) and Sticks to teeth (5%) were included due to their expected relevance for sample discrimination in the set, and (iii) terms Looks unhealthy (3%) and Whole wheat/grain (2%) were included despite their low frequencies as they were deemed interesting for the study. In total, 18 terms were selected and classified into appearance (*n* = 5), texture (*n* = 8) and taste/flavour terms (*n* = 5) (Table 2). Children used few terms for smell and those were related to taste/flavour terms, therefore it was decided not to include specific smell terms. Finally, the selected terms were back-translated into the local languages, with attention paid to preferably using a word or phrase originally elicited by children where possible, in order to ensure child-friendly vocabulary for the age group in each country.

### 2.4. Child Food Neophobia Scale

The children completed the child food neophobia scale [12]. Eight items were scored on a 5-point facial scale ranging from ‘very false’ to ‘very true’. This questionnaire was originally developed in Italy but was for the purpose of this study translated to the local languages. The reliability and validity of the scale in the different countries are reported elsewhere [13]. A food neophobia score was calculated for each child by summing the answer to every item after reversing the neophilic ones. The total score ranged from 8 to 40 where a higher score implies a higher degree of food neophobia, see data analysis.

### 2.5. Procedures

Data were collected in two sessions, either on two different days or on the same day with a break in-between sessions. The tests were conducted in schools or nearby facilities. Children were either tested individually in a room or in a class setting in groups from 12 to 25 children at a time.

In the first session, children answered a web-based questionnaire using tablets. This questionnaire included the Child food neophobia scale, questions on food texture preferences (reported in Proserpio et al. [13], Laureati et al. [33]) and questions about gender, age as well as two general questions related to biscuits: “Do you like biscuits”? (Three-point smiley scale: Yes, It’s ok, No) and “How often do you eat biscuits?” (never, every month, every week, every day or almost every day, only on special occasions, other, I don’t know).

In the second session, children were asked to monadically take a small bite of the eight biscuits in a balanced random order. They were asked: “How much do you like this biscuit?” and rated their acceptance through a 7-point horizontal facial hedonic scale with three anchors (I do not like it at all, I neither like it nor dislike it, I like it very much). After tasting each biscuit, they were asked to perform the CATA task “Choose all the words that describe the biscuit”. The terms presented were categorized by appearance, texture and taste/flavour. The terms were randomized within each modality and across subjects, but not within subject. The children were asked on screen to drink a sip of water and thereafter proceed with the next biscuit. After all the eight biscuits had been tasted, the children were asked to think about their ideal imaginary biscuit, and similarly to the tasted biscuits, to rate acceptance and describe it using the CATA terms. The protocol, shared by all countries, included specific instructions for the children, such as that there were no wrong or correct answers and that the experimenters were interested in their own opinion and perception related to the biscuits. The children were not aware of the content and different fibre levels of the biscuits. Both sessions together took on average 33 min to conduct.

A web-based questionnaire was used to collect data from parents. One adult per child could answer. Parents provided data on the child’s birth country and area of living (large city/medium town/small town or rural area), as well as their perceived economic situation on a 7-point scale (“1 = difficult, “4 = moderate” and “7 = well-off”) [34] and parental educational level.

### 2.6. Data Analysis

The frequency distribution of food neophobia scores was calculated over all countries and by country. According to Shapiro-Wilks test, the distribution in the overall sample deviated from normal distribution (*p* = 0.026). Investigation of the Q-Q plot however showed a normal pattern and thereby the data were handled as normally distributed. Country-wise, only Spain deviated from normality according to Shapiro-Wilks test (*p* = 0.011). The 25% and 75% quartiles of the child food neophobia scale over all countries were used to segment the children into three food neophobia status groups: one neophilic group-with lower degree of food neophobia (scores ≤ 17, *n* = 144), one neophobic group-with higher degree of food neophobia (scores ≥ 24, *n* = 142) and a medium neophobia group (scores 18–23, *n* = 223).

Liking of the eight biscuits was examined using 3-way Analysis of Variance (ANOVA) considering samples (eight biscuits), country (Finland, Italy, Spain, Sweden and UK), neophobia status (low, medium, high) and their interactions as factors. Due to a large country-related variation in biscuit consumption frequency, this was added as a factor in a subsequent model. This analysis excluded children who had answered “never” (1.2%), “other” (4.3%) and “I don’t know” (16.7%) on biscuit consumption.

The total mean use of CATA terms per biscuit was calculated as well as the total mean use of specifically appearance, texture and taste/flavour terms. According to Shapiro-Wilks tests, the distribution of total CATA terms in the overall sample displayed a normal distribution (*p* = 0.117), while the specific sensory modalities showed a non-normal distribution. Investigation of the Q-Q plot however showed normal patterns so the data were considered normally distributed. Pearson 2-tailed correlations were used to explore associations between degree of neophobia and the total use of CATA-terms. Thereafter, 2-way ANOVAs with the factors country, neophobia status and their interaction were applied. Note that preliminary analyses showed that gender was not significant, and this factor was therefore not further investigated. Bonferroni test was used for post-hoc analyses.

Cochran’s Q test was performed for each of the 18 terms to evaluate differences between the biscuits, i.e., if children used the terms to differentiate between the biscuits. This was done for the whole sample, separately according to neophobia level (low, medium, high), as well as for each country.

Principal coordinate analysis and penalty analysis were used to study the association between the liking scores of the eight tasted biscuits and their CATA-descriptions. By analysing CATA-term occurrences in light of ideal product descriptions and liking evaluations, the penalty analysis classifies attributes as “must have” (i.e., the attribute is required to get higher liking score and for the ideal product), “nice to have” (i.e., the attribute is positive for liking, but not required in ideal product), “does not influence” (i.e., the attribute has no effect on liking and is not a must have), “does not harm” (i.e., the attribute has no effect on liking and is not required in ideal product) and “must not have” (i.e., the attribute lowers liking scores and is not required in ideal product) [35,36]. These analyses were performed for all participants as well as separately according to neophobia level (low, medium, high) and for each country.

Chi-square analyses and ANOVA were used to examine if background variables varied by food neophobia groups. In significance tests, p-values below 0.05 were considered significant. IBM SPSS Statistics 24 (IBM Corp, Armonk, NY, USA) and XLSTAT version 2019.1.2 (Addinsoft, Paris, France) were used to perform the analyses.

## 3. Results

### 3.1. Description of the Participating Children

In total, 509 children from Finland, Italy, Spain, Sweden and UK were included in the analysis. The mean age was 10.4 (SD 0.7) years and 54.6% were girls (Table 3). The mean food neophobia score in the total sample was 20.6 (SD 5.3). In total, 28.3% of the children were classified as food neophilic i.e., with a low degree of neophobia, 43.8% with a medium degree and 27.9% as food neophobic i.e., with a high degree of neophobia. With regard to the general question, “Do you like biscuits?” 91.2% of the children answered “yes”, 8.4% “it’s ok” and 0.4% “no”. Chi-square analysis showed no significant differences for the questions: “Do you like biscuits?” and “How often do you eat biscuits” with regard to neophobia status. On average 65% (*n* = 332) of the parents completed the parental questionnaire. The majority of the children were born in the respective country of residence and lived in a large city. Children in large cities were more often classified as neophilic and children in rural areas were more often classified as neophobic (*p* = 0.008). Among 72.5% of the children, one or both parents had a university degree. The perceived economic status was on average moderate or high. Country-related differences were found with regard to reported consumption of biscuits (*p* < 0.001). A high percentage of the children in Italy (57.6%) and Spain (41.4%) reported eating biscuits every day or almost every day, while this was somewhat less common in the UK (24.2%) and very uncommon in Sweden (8.4%) and Finland (2.9%).

### 3.2. Biscuit Liking

Mean liking on the 7-point scale, over all biscuits and all children was 5.4 (SD = 1.65). A 3-way ANOVA showed significant effects of the main factors Biscuit (*p* < 0.001), Neophobia status (*p* < 0.001) and Country (*p* < 0.001), as well as an interaction between Biscuit and Country (*p* < 0.001). Post-hoc tests showed that all three neophobia status groups differed significantly in total liking. Food neophilic children displayed the highest degree of liking for all biscuits (mean liking 5.7; SD = 1.5) and food neophobic children displayed a lower degree of liking than the other two groups (mean liking 5.0; SD = 1.8). Children in the UK displayed a significantly lower degree of liking compared to the other countries. Overall, the Classic wheat biscuit (mean liking 5.9; SD = 1.5) and the Chocolate chip biscuit (5.8, SD = 1.5) were the most liked and the Dried fruit biscuit (5.1; SD = 1.7) together with the Apple jam biscuit the least liked (5.0; SD = 1.9).

When adding consumption frequency to the model (only including children who answered every month, every week, every day, and on special occasions, *n* = 396) the Country*Biscuit interaction was no longer significant. Consumption frequency was a significant main factor (*p* = 0.001) as well as the two-way interactions with country (*p* = 0.003) and neophobia (*p* = 0.014). Further, a three-way interaction was found between country*neophobia*consumption (*p* = 0.001). Children reporting to consume biscuits everyday displayed a higher degree of overall liking compared to those reporting to eat on special occasions and every week. However, in Italy, children who reported to eat only on special occasions displayed the highest liking. In Sweden, Finland and Spain, children with a high degree of neophobia who reported to only eat biscuits at special occasions displayed the lowest degree of liking.

### 3.3. Use of CATA-Terms

Per biscuit, the children used on average 6.0 (SD = 2.1) CATA-terms out of 18 to describe the samples. Significant negative correlations were found between the degree of neophobia and the total use of CATA terms (*r* = −0.116, *p* = 0.009), the use of appearance terms (*r* = −0.129, *p* = 0.004) and the use of taste/flavour terms (*r* = −0.088, *p* = 0.047) while the correlation with texture was not significant (*r* = −0.087, *p* = 0.051). With regard to the specific CATA-terms, a higher degree of food neophobia was associated with a less frequent use of six of the 18 terms. These were the appearance terms ‘tempting’ and ‘whole wheat’, texture terms ‘crunchy’, ‘grainy’ and ‘smooth’ as well as the taste term ‘sweet’ (Table 4). Table 4 also shows the correlation between degree of neophobia and use of CATA-terms in the specific countries. One exception to the lower usage of CATA terms was found in Sweden where neophobia was associated with a higher frequency of the texture term ‘dry’ (*r* = 0.186, *p* = 0.043).

The 2-way ANOVAs using segmentation of the children into the three neophobia status groups showed no significant main effect of food neophobia status in the use of CATA-terms. Country was a significant main factor for the use of appearance attributes (*p* = 0.002). A post-hoc test showed that children in Spain used significantly more appearance terms compared to children from Sweden and the UK. No 2-way interaction was found between neophobia status and country.

Among all the biscuits and the 18 included terms, children made use of a mean of 14.8 (SD = 2.7) terms, ranging from 3–18 terms. No difference in neophobia status was found, but a country difference was present (*p* = 0.001). Bonferroni post-hoc test showed that children in Finland used significantly more terms (mean 15.8, SD = 1.7, min 11, max 18) compared to children in Sweden (mean 14.2, SD = 2.9, min 3, max 18) and Italy (mean 15.5, SD = 2.5, min 5, max 18).

Further, Cochran’s Q test for each CATA term showed that the terms did generally significantly discriminate between biscuits, within each of the three neophobia status groups, with the exceptions of the texture attribute ‘sticks to teeth’ among neophobics and neophilics as well as ‘crumbly’ in neophobics. Specifically, in each country, the texture attribute ‘sticks to teeth’ did not discriminate the different samples in Spain and the UK, and the texture attribute ‘crumbly’ did not discriminate the different samples in Italy.

### 3.4. Drivers of Biscuit Liking

**Figure 1:**
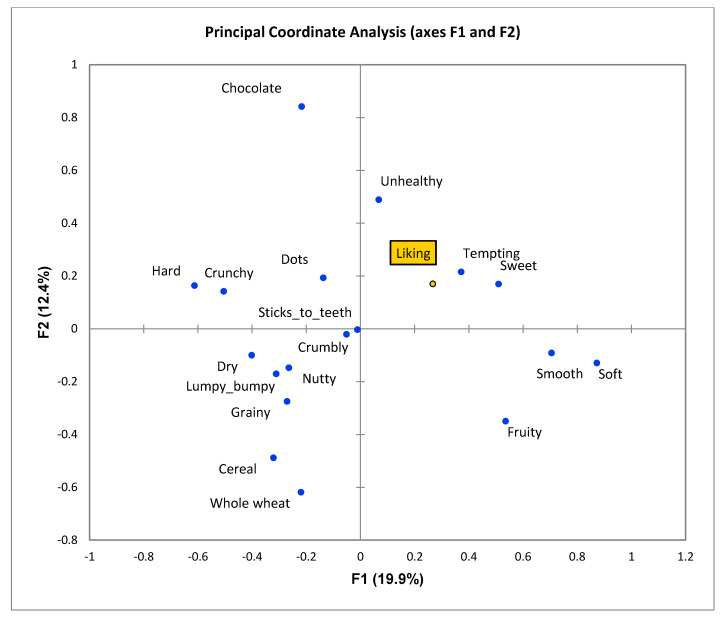
Principal coordinate plot based on CATA descriptions and liking of eight biscuits for the whole sample of children (*n* = 509).

**Figure 2:**
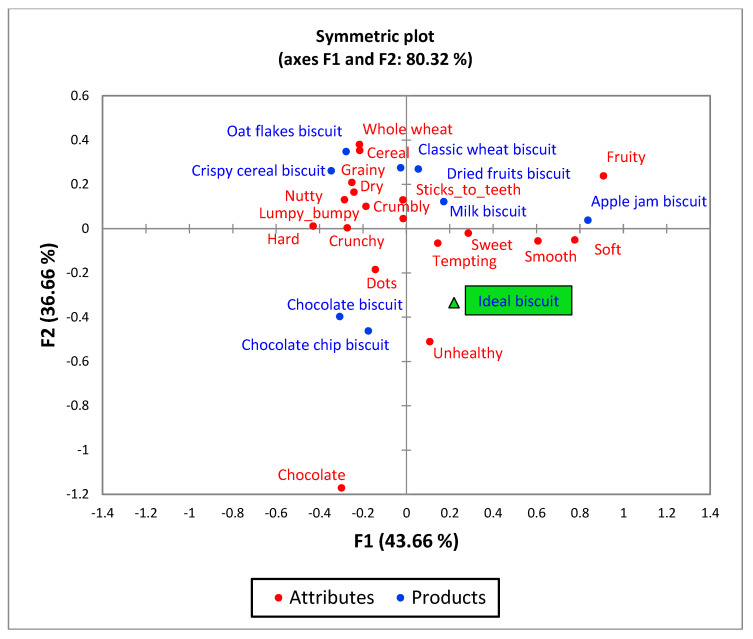
Symmetric plot based on CATA descriptions of 8 biscuits as well as of the children’s ideal biscuit for the whole sample of children (*n* = 509).

Principal Coordinate Analysis, linking liking to CATA descriptions showed similar results both among different neophobia status groups as well as between the countries. Thus, a plot for the whole sample is shown here, where axes F1 and F2 represent the first two latent dimensions of the model (Figure 1). It displays liking to be positively associated with the CATA terms ‘sweet’ (taste) and ‘tempting’ (appearance) and negatively related to ‘whole wheat’ (appearance) and ‘cereal’ (taste). The relatively low proportion of variance explained by the first two dimensions (32.4%) testifies of large individual variations in biscuit liking across children. In line with the liking results, the symmetric plot based on the CATA description of the eight real biscuits as well as an imaginary ideal biscuit (Figure 2) shows that an ideal biscuit is typically characterised by attributes ‘unhealthy’, ‘soft’, ‘smooth’, ‘tempting’ and ‘sweet’, and not by attributes ‘whole wheat’, ‘cereal’ and ‘grainy’. None of the test samples fall in the ideal direction, with the two chocolate biscuits being closest, but too hard to be ideal. In this analysis, the high proportion of explained variance on the first two factors (80.3%) testifies of a good agreement in sample descriptions across children.

A compilation of results from the penalty analyses performed for the different groups of children per neophobic level and per country is found in Table 5. Over all children, the must-have attributes in a biscuit are: a ‘tempting’ appearance, a ‘crunchy’ texture that is ‘soft to bite’ and ‘smooth in mouth’, taste ‘sweet’ and flavour ‘chocolate’. Must-not-haves are appearance with ‘dots’ and a ‘dry’ texture that ‘sticks to teeth’. Comparing children with different degrees of neophobia, the main differences are found in the appearance attributes. For neophobics, the biscuit must not look ‘lumpy or bumpy’ or look like it contains ‘whole wheat’. Country-wise differences are also revealed. For example, while a ‘crunchy’ texture is a “must have” in Sweden and Spain, it is a “must not have” in UK. Italy does not show any particular texture-related preferences. All countries agree on ‘sweet taste’ being a “must have”, and all countries except Finland included ‘chocolate’ as a “must have”.

## 4. Discussion

The aim of this paper was to explore differences in liking and sensory perception of fibre-rich biscuits in a cross-cultural sample of children with different degrees of food neophobia. Using the CATA method, the paper investigated how neophobic status and/or cultural background may result in a different usage of sensory descriptors and in different drivers of liking and disliking. Children with a higher degree of neophobia generally displayed a lower liking for all tasted biscuits. Cross-cultural differences in liking were also found and seemed to be related to the difference in consumption frequency of biscuits between countries. The results indicated that children with a higher degree of neophobia used fewer CATA terms to describe the samples, particularly for the description of appearance and taste/flavour but not for texture. With regard to drivers of liking, degree of neophobia affected particularly appearance and textural attributes while no differences were found for taste and flavour attributes.

The finding that food neophobic children used fewer CATA terms than neophilic children to describe the samples is interesting and deserves further explanation. Literature based on large population studies in adults has shown that food neophobics perceive more intensely than neophilics “warning” sensations such as bitterness, astringency and pungency, which are signals of potentially toxic and/or unpleasant food [17,37]. This has been explained as a consequence of their increased alertness during food consumption possibly due to the higher anxiety state related to the meaning, rather than the intensity, of the sensory stimuli [17,37]. Similar conclusions have been put forward also in studies involving children [15,16]. These findings suggest a more cautious and, presumably, a more analytical approach to food in neophobic children compared to neophilic children. In the present study, food neophobic children used the terms ‘sweet’, ‘smooth’, ‘grainy’, ‘crunchy’ and ‘looks tempting’ less often than their food neophilic peers. One possible explanation is that biscuits are not considered as a potentially dangerous or unpleasant food; they are usually characterised by sensory properties that are expected to be liked, especially by children. This might have resulted in a lower psychological arousal and a lower alertness state by neophobic children compared to other foods [38]. Moreover, people scoring high in food neophobia are possibly not only those who have a fear of new foods; they may also be individuals who have little interest in foods [39] and less positive associations with food throughout their lives [40]. The fact that the terms that were less used by neophobic children, had in general a positive connotation (e.g., sweet, tempting) may indeed support this hypothesis. Finally, familiarity with the product plays an important role in vocabulary richness [41]. Although biscuits are generally a well-known product for children, food neophobics’ more limited exposure to food may partially explain their poorer sensory vocabulary. Further research is needed to confirm and expand these findings possibly by exploring food neophobia-related differences in the description/perception of pleasant vs. unpleasant food or food characterised by warning sensations.

In addition, food neophobic children displayed a lower liking than the food neophilic children for the tasted biscuits. The pattern was similar in all countries, despite the biscuits being available in the Italian market. Previous literature reports that it’s unclear whether a lower degree of liking per se is coupled to a lower use of descriptive terms [42]. Although some sensory properties were liked and desired in whole-wheat biscuits overall (i.e., sweet taste and tempting appearance), we found that drivers of rejection varied according to neophobia level, especially with reference to appearance. Children with higher food neophobia rejected biscuits that had a lumpy or bumpy appearance and looked like whole-wheat. This finding is in line with previous literature indicating that food rejections in children primarily occur on sight and may be related to visually perceived texture [18]. Similar results were found for orally perceived texture, with children rejecting apple puree [29], vegetables [43] and yoghurt [44] with a granular, non-uniform structure, suggesting that the presence of particles may be a deterrent to the consumption of healthy food in young consumers. In this context, it is important to improve the formulation of products that contain fibre in such a way as to reduce the negative impact it gives to the appearance of the product and limit the refusal by neophobic children. In terms of recommendations to food producers, our results show that when it comes to fibre-rich biscuits, neophobic children prefer biscuits with chocolate aroma, which may be convenient to visually dissimulate whole grain flours. Besides this constraint, many aroma and texture attributes were accepted (read: not rejected) by our neophobic participants, offering opportunities for healthier product formulations. These were fruity, cereal and nutty aromas, as well as texture attributes crunchiness, crumbliness, hardness, softness, dryness, graininess, smoothness and stickiness. Indeed, focusing on product formulation seems to be the way to reach out to neophobics also into adulthood. Based on different studies in adults, Jaeger and collaborators [45] report attributes ‘familiarity’, ‘convenience’ and ‘sensory appeal’ as especially important factors in food neophobics’ food choices, while attributes ‘health’, ‘natural content’, and ‘environmental’ and ‘social justice’ concerns decrease in importance with higher neophobia. Thus, while credence attributes play an important role in food neophilics’ choices, mostly search and experience attributes [46] seem to matter in food neophobics’ choices.

The present study has also relevance from a methodological point of view as we used the CATA approach to compare not only children with different neophobia levels but also from different countries. Recently, the CATA approach has been successfully used with sensory [29,47,48,49,50,51], emotional [50,51,52,53] and hedonic attributes [54] to investigate children’s perception and to get insights on properties that children perceive and consider desirable or undesirable in food. Only one of these studies did a cross-cultural comparison among children in their liking and perception of fruit juices [47] but since this was not the main aim of the study, unfortunately, country-related differences, when found, were not commented, therefore comparison with the present study is not possible.

Cross-cultural research is becoming increasingly relevant in sensory and consumer science. A better understanding of whether and how differences in the food environments and dietary experiences across cultures influence food preference, choice, attitudes and beliefs is important for food product development [41]. However, cross-country research with children is very limited. In this context, in the present study, important country-related differences in the consumption, liking and drivers of (dis)liking of high-fibre biscuits were highlighted. Drivers of liking for all countries were expected sensory properties such as sweetness and chocolate taste, whereas dry texture seemed a driver of rejection for almost all countries. Great cross-country variability in texture drivers was found. In particular, while crunchiness was a must-have in Spain and Sweden, it was identified as a must-not-have in the UK, and as an indifferent attribute in Italy and Finland. More generally, while the Spanish subjects strongly favoured or rejected five out of the eight texture attributes, the Italian subjects showed no particular preference or rejection for any of these.

A strength of this study was the cross-cultural design and that the same sensory terms were used for all countries, but this also includes challenges [41]. Large country differences were found in biscuit consumption. Use of the textural terms ‘Sticks to teeth’ and ‘crumbly’ did not differ significantly between the biscuits in some countries. The vocabulary used to describe sensory experiences strongly depends on culture and previous exposure to different product experiences [41] which might explain these results. However, when checking usage of the attributes, we found that in all countries and across all samples, some children used all 18 available CATA attributes, which indicated that the attributes were very applicable [48].

A limitation of the present study is that although the Food Neophobia Scale used has been validated in all the included countries, these results have not yet been confirmed with actual behavioural measurements [13]. The present study however provided evidence of differences in sensory perception and liking between children with different levels of food neophobia. Moreover, the biscuits were available on the Italian market although targeted to adults. Notwithstanding similar biscuits are available in all countries, it cannot be excluded that familiarity with the product, which was not assessed in the present study, may have played a role on the outcome. The difference in consumption frequency of biscuits between the countries may however indicate that children in Italy, Spain and UK have a higher familiarity with biscuits in general compared to Sweden and Finland.

Also, important to consider is that what we eat is dependent on many factors, including biological (e.g., sex, genetics) and environmental variables (e.g., parental eating behaviour and lifestyle), considering all these factors in a single study is difficult. In the present study, we mainly focused our attention on two factors: food neophobia and country of residence. Controlling for socio-demographic variables might be particularly important in cross-national studies [41]. In the present study, data on gender, birth country, area of living, economic situation and parental educational degree were included to describe the sample. Preliminary results indicated no effect of gender on usage of CATA-terms. However, potential gender differences should be explored in future research. Degree of food neophobia varied with living area among the children in the present study. This is in line with previous large-scale studies on adults in different countries showing food neophobia to be lower in large cities [39,55]. Inhabitants of a rural area may have fewer opportunities to be exposed to new foods. Results from the present study indicate that these differences are displayed already in 9–12-year-old children.

Considering the well-known positive effects of fibre on human health and that fibre intake is below the recommended levels in all Western countries [56], trying to guide children toward a higher consumption of whole-grain and fibre-rich foods is certainly a challenge for nutritionists and food companies [57,58]. One way is to develop alternative versions of familiar products. However, for these products to have a potential health effect, they need to be available, chosen and eaten by consumers [59]. Further research is needed to understand the inter-relationship between the main factors involved in determining dietary behaviours. The present study shows how sensory properties affect children’s liking of fibre-rich biscuits taking into consideration individual levels of food neophobia and country of residence. Further research should explore if optimizing appearance attributes could be a way to increase liking of fibre-rich foods in neophobic children and if these findings could be applied to other foods and to home cooking, emphasizing the importance of visual attributes when changing a recipe or serving new foods. Alternatively, giving the child time to familiarize themselves with the visual attributes before having to taste [60].

## 5. Conclusions

The present study shows that the degree of food neophobia affects how the sensory attributes of food are perceived and evaluated in 9–12-year-old children. Food neophobic children used fewer CATA terms to describe fibre-rich biscuit samples, and several appearance properties were found to be drivers of disliking in biscuits for this group. Cultural differences also played a role, especially in the use of appearance attributes to describe the biscuits and in the preference or rejection of texture properties across countries. From a methodological perspective, the present results emphasize the necessity of being cautious when generalizing results to different countries as well as the importance of taking into consideration individual factors such as food neophobia when interpreting results from sensory studies with children. Considering that food perception strongly influences food preference and consumption, a better understanding of the drivers of (dis)liking of fibre-rich food among vulnerable populations such as children might be helpful from a public health perspective. The optimization of the sensory properties of healthy food is a key strategy to improve its liking and promote its consumption even among children with high levels of food neophobia [61]. In this context, findings of the present study may be useful for food practitioners to develop healthy alternative formulations of familiar foods that are well accepted by children, with the aim of promoting consumption especially among neophobic children, which can be more at risk of developing nutritional deficiencies.

## Figures and Tables

**Table 1:** Main ingredients, fibre and sugar content of the eight biscuits included in the study.

Biscuit	Main Ingredients	Fibre Content (g/100g) *	Sugar Content (g/100g)
Apple jam	Wheat flour, apple jam, sunflower oil, sugar	2.8	31.0
Milk	Wheat flour, sugar, sunflower oil, wheat starch, skim milk powder	3.4	21.6
Classic wheat	Wheat flour, sugar, whole wheat flour, sunflower oil	4.6	22.0
Crispy cereals	Crispy cereals, oat flakes, sunflower oil, sugar	6.5	15.5
Oat flakes	Wheat flour, sunflower oil, oat flakes, sugar	8.0	15.5
Chocolate	Wheat flour, sugar, oat flakes, caramelized hard wheat, chocolate	9.1	29.5
Dried fruits	Whole wheat flour, oats flakes, sunflower oil, sugar, whole spelt, cranberries, hazelnuts, apples	9.5	20.6
Chocolate chip	Wheat flour, chocolate, sugar, sunflower oil, oatmeal flour	10.0	20.0

* ≥3 g of fibre per 100 g allows for using the nutritional claim “source of fibre”, ≥6 g of fibre per 100 g allows for the use of the nutritional claim “high in fibre” according to European regulation 1924/2006.

**Table 2:** Included CATA-terms according to sensory modality.

Sensory Dimension	English	Finnish	Swedish	Italian	Spanish
**Appearance**	Lumpy or bumpy	Paakkuinen tai rakeinen tai epätasainen	Buckligt eller ojämnt	Superficie irregolare (non liscia)	Superficie irregular (no lisa)
	I see dots/spots	Näen pilkkuja tai täpliä tai erilaisia värejä	Jag ser prickar	Ha puntini/macchioline	Tiene puntos/manchas
	Looks tempting	Näyttää houkuttelevalta	Ser frestande ut	Sembra invitante/gustoso	Parece sabrosa/apetecible
	Looks unhealthy	Näyttää epäterveelliseltä	Ser onyttigt ut	Sembra poco sano	Parece poco sana
	Whole wheat/grain	Täysjyvä	Fullkorn	Fatto con farina integrale	Hecha con harina integral
**Texture**	Crunchy	Rapea	Knaprigt	Croccante	Crujiente
	Crumbly	Mureneva (rikkoutuu palasiksi, kun puraiset sitä)	Smuligt	Si sbriciola facilmente	Se rompe fácilmente
	Hard to bite	Kova purra	Hårt att bita i	Duro da mordere	Dura al morder
	Soft to bite	Pehmeä purra	Mjukt att bita i	Morbido quando lo morsico	Blanda al morder
	Dry (Makes you thirsty)	Kuiva (tekee sinut janoiseksi)	Torrt	Secco	Seca
	Grainy	Rakeinen	Grynigt	Granuloso	Granulosa (arenosa en boca)
	Smooth in mouth	Tasainen ja sileä suussa	Lent i munnen	Liscio in bocca	Suave en boca
	Sticks to teeth	Tarttuu hampaisiin	Fastnar i tänderna	Si attacca ai denti	Se pega a los dientes
**Taste/Flavour**	Sweet taste	Makea	Smakar sött	Dolce	Dulce
	Fruity taste	Hedelmäinen maku	Smakar fruktigt	Sa di frutta	Sabor a fruta
	Cereal taste	Viljainen maku (kaura, vehnä, maissihiutaleet, jauho)	Smakar spannmål (havre, vete, mjöl)	Sa di cereali	Sabor a cereales
	Chocolate taste	Suklaan makuinen	Smakar choklad	Sa di cioccolato	Sabor a chocolate
	Nutty taste	Pähkinäinen maku	Smakar nötter	Sa di nocciole	Sabor a nueces

**Table 3:** Background characteristics and food neophobia status of the participating children.

		Food Neophobia Status *		
	All	Neophilic	Medium	Neophobic
N	509	144	223	142
%		28.3	43.8	27.9
Gender (% girls)	54.6	53.5	55.2	54.9
Age in years (mean ± SD; range)	10.4 ± 0.7 (9–12)	10.5 ± 0.7 (9–12)	10.4 ± 0.7 (9–12)	10.4 ± 0.7 (9–12)
Country (%)				
Finland (*n* = 70)	13.8	16.7	13.0	12.0
Italy (*n* = 85)	16.7	22.9 ^b^	17.5 ^b^	9.2 ^a^
Spain (*n* = 111)	21.8	23.6	23.3	17.6
Sweden (*n* = 119)	23.4	16.7 ^a^	22.9 ^a,b^	31.0 ^b^
UK (*n* = 124)	24.4	20.1 ^a^	23.3 ^a,b^	30.3 ^b^
Born in country of residence (%) **	94.0	97.8	92.4	92.7
Area of living (%) **				
Large city or municipality near large cities	65.1	73.6 ^b^	59.0 ^b^	51.0 ^a^
Medium sized town or municipality near medium sized towns	26.8	23.1 ^a^	22.8 ^a^	36.5 ^b^
Smaller town, smaller urban area or rural municipality	8.1	3.3 ^a^	8.3 ^a,b^	12.5 ^b^
Family economic situation (mean ± SD) ** ‡	5.0 (1.5)	5.0 (1.3)	5.0 (1.3)	5.1 (1.4)
Parent with university degree (%) **	72.5	76.9	70.7	70.8
Do you like biscuits? (%)				
No	0.4	0.7	0.0	0.7
It’s ok	8.4	7.6	8.1	9.9
Yes	91.2	91.7	91.9	89.4
How often do you eat biscuits? (%)				
Never	1.2	0.7	0.9	2.1
Every month	13.0	10.4	13.0	15.5
Every week	22.4	22.2	23.3	21.1
Every day or almost every day	26.9	31.3	24.7	26.1
Only on special occasions	15.5	18.1 ^b^	17.9 ^b^	9.2 ^a^
Other	4.3	6.9	3.6	2.8
I don’t know	16.7	10.4 ^a^	16.6 ^a,b^	23.2 ^b^
Food neophobia (mean; SD; range)	20.6; 5.3 (8–37)	14.3; 2.4 (8–17) ^a^	20.5; 1.6 (18–23) ^b^	27.5; 2.9 (24–37) ^c^

* Food Neophobia was measured with the Child Food Neophobia Scale, ranging in total score from 8–40. The 25% and 75% quartiles in Food Neophobia score were used for segmentation into food neophilic (scores ≤ 17), medium food neophobia (scores 18–23) and food neophobic (scores ≥ 24) ** Based on parent reports from *n* = 332 children. ^a,b^ Different superscript letters indicate significant differences between food neophobia groups. ‡ Measured on a 7-point scale: 1 = difficult, 4 = moderate, 7 = well-off.

**Table 4:** Pearson correlations between degree of food neophobia and use of CATA terms in eight biscuits, for the total sample of children and per country.

	Total Sample (*n* = 509)	FI (*n* = 70)	IT (*n* = 85)	ES (*n* = 111)	SE (*n* = 119)	UK (*n* = 124)
Total CATA terms	−0.116 **	−0.120	−0.260 *	−0.112	0.026	−0.171
Appearance	−0.129 **	−0.159	−0.250 *	−0.106	−0.035	−0.134
Lumpy or bumpy	−0.068	−0.122	−0.314 **	−0.090	0.103	0.017
I see dots/spots	−0.026	−0.113	0.053	−0.041	0.125	−0.124
Looks tempting	−0.161 **	−0.200	−0.192	−0.086	−0.103	−0.136
Unhealthy	0.009	0.097	−0.190	0.000	0.018	−0.063
Whole wheat/grain	−0.100 *	−0.080	−0.023	−0.104	−0.224 *	−0.081
Texture	−0.087	−0.085	−0.241 *	−0.113	0.045	−0.138
Crunchy	−0.088 *	−0.267 *	−0.168	−0.126	0.022	−0.054
Crumbly	0.002	0.014	−0.105	−0.104	0.081	−0.018
Hard to bite	0.006	0.014	−0.044	0.013	0.020	−0.045
Soft to bite	−0.064	0.118	−0.055	−0.034	−0.102	−0.159
Dry	−0.001	−0.039	−0.096	−0.088	0.186 *	0.009
Grainy	−0.131 **	−0.031	−0.153	−0.080	−0.145	−0.154
Smooth in mouth	−0.119 **	−0.208	−0.032	−0.061	−0.117	−0.195 *
Sticks to teeth	−0.007	0.034	−0.288 **	−0.003	0.115	−0.046
Taste/Flavour	−0.088 *	−0.086	−0.155	−0.052	0.045	−0.199*
Sweet	−0.142 **	−0.323 **	−0.177	−0.044	0.044	−0.264 **
Fruity	−0.064	0.192	−0.147	−0.226 *	0.086	−0.169
Cereal	−0.057	−0.104	−0.048	0.078	−0.053	−0.032
Chocolate	−0.024	0.199	−0.042	−0.036	0.067	−0.191 *
Nutty	0.066	0.082	0.017	−0.002	0.047	0.032

* Correlation is significant at the 0.05 level (2-tailed); ** Correlation is significant at the 0.01 level (2-tailed); FI: Finland, IT: Italy, ES: Spain, SE: Sweden, UK: United Kingdom.

**Table 5:** Drivers of liking and disliking identified by penalty analysis.

	All N = 509	Neophobia Status			Country				
		Neophilic N = 144	Medium N = 223	Neophobic N = 142	FI N = 70	IT N = 85	ES N = 111	SE N = 119	UK N = 124
Appearance									
Lumpy or bumpy	-	-	-	X	X	-	-	-	X
I see dots/spots	X	-	X	-	○	-	-	-	-
Looks tempting	√	√	√	√	√	√	√	√	√
Looks unhealthy	-	-	-	-	-	-	-	n.s.	-
Whole wheat/grain	X	-	-	X	-	-	-	X	-
Texture									
Crunchy	√	√	√	○	○	○	√	√	X
Crumbly	-	-	-	n.s.	-	n.s.	-	-	-
Hard to bite	-	-	-	-	-	-	X	-	-
Soft to bite	√	√	√	○	√	○	-	○	○
Dry	X	X	X	-	X	-	X	X	X
Grainy	-	X	-	-	-	-	X	-	-
Smooth in mouth	√	√	√	-	√	-	√	-	√
Sticks to teeth	X	n.s.	X	n.s.	X	-	n.s.	-	n.s.
Taste/flavour									
Sweet	√	√	√	√	√	√	√	√	√
Fruity	-	-	-	-	-	-	-	-	-
Cereal	-	-	-	-	-	X	-	-	-
Chocolate	√	√	√	√	○	√	√	√	√
Nutty	-	-	-	-	-	-	-	-	X

n.s.: non-significant attribute. √: Must have; ○: Does not influence; -: Does not harm; X: Must not have. None of the attributes were classified as nice-to-haves. FI: Finland, IT: Italy, ES: Spain, SE: Sweden, UK: United Kingdom.

## Data Availability

The data presented in this study are available on request from the corresponding authors.
